# Isolation of Epstein‐Barr virus‐deoxyribonucleic acid in the lower respiratory tract for distinguishing critically ill patients from those with influenza‐associated pneumonia: A pilot study

**DOI:** 10.1111/crj.13710

**Published:** 2023-10-12

**Authors:** Yu Bai, Yinqun Guo, Xi Chen, Chunxia Yang, Li Gu

**Affiliations:** ^1^ Department of Infectious Diseases and Clinical Microbiology, Beijing Institute of Respiratory Medicine and Beijing Chao‐Yang Hospital Capital Medical University Beijing China

**Keywords:** ARDS, Epstein–Barr virus, influenza, pneumonia

## Abstract

**Background:**

When influenza A‐related pneumonia is complicated by bacteria, aspergillus, and other infections, the disease is aggravated, while there is no research on the role of Epstein–Barr virus (EBV) on patients with influenza A‐related pneumonia. This study aimed to evaluate the relationship between the isolation of EBV and influenza A‐related pneumonia.

**Methods:**

This is a clinical study based on the baseline data of a retrospective cohort. A total of 113 cases of influenza A‐related pneumonia who underwent polymerase chain reaction test for isolation of EBV‐DNA in lower respiratory tract specimens during six influenza seasons from 2013–2014 to 2018–2019 were enrolled. According to the results of EBV‐DNA, patients were divided into EBV‐positive group and EBV‐negative group, and the role of EBV‐DNA on patients with influenza A‐related pneumonia was analyzed. Regression analysis was used to explore the potential risk factors for the development of moderate‐to‐severe acute respiratory distress syndrome (ARDS) in patients with influenza A‐related pneumonia during hospitalization.

**Results:**

Among 113 patients with influenza A‐related pneumonia, there were 53 patients with EBV‐positive and 60 patients with EBV‐negative. The EBV‐positive group had higher intensive care unit admission rate, hospital stay, invasive mechanical ventilation rate, extracorporeal membrane oxygenation rate, Sequential Organ Failure Assessment (SOFA) score, and moderate‐to‐severe ARDS rate. Patients were divided into severe group and mild group. Patients in severe group had lower lymphocyte count, platelet count, and albumin level, while the levels of aspartate aminotransferase, alanine transaminase, creatine kinase, lactic dehydrogenase, and total bilirubin were higher in severe group. The levels of D‐dimer, serum ferritin, C‐reactive protein, and procalcitonin were higher in severe group than those in the mild group. Multivariate logistic regression analysis revealed that the isolation of EBV (odds ratio = 2.713, 95% confidence interval: 1.094–6.729, *P* = 0.031) and lymphocyte count (odds ratio = 3.585, 95% confidence interval: 1.157–11.101, *P* = 0.027) were independent risk factors for moderate‐to‐severe ARDS in patients with influenza A‐related pneumonia.

**Conclusion:**

The isolation rate of EBV in the lower respiratory tract was 46.9%. The length of hospital stays, intensive care unit admission rate, invasive mechanical ventilation rate, extracorporeal membrane oxygenation rate, SOFA score, and the proportion of moderate‐to‐severe ARDS in the EBV‐positive group were higher than those in the EBV‐negative group, while there was no effect on the death during hospitalization. The isolation of EBV in the lower respiratory tract and low lymphocyte count are independent risk factors for the development of moderate‐to‐severe ARDS in patients with influenza A‐related pneumonia.

## Graphical abstract

The detection rate of EBV‐DNA in the lower respiratory tract was high. When patients have positive EBV‐DNA results in the lower respiratory tract, their disease is often more severe. EBV‐DNA‐positive and low lymphocyte count are independent risk factors for the development of moderate‐to‐severe ARDS in patients with influenza A‐related pneumonia.

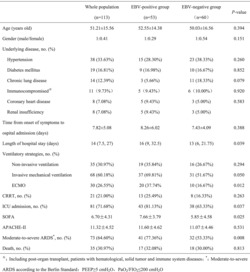

**Abbreviations**
EBV: Epstein–Barr virus
ECMO: extracorporeal membrane oxygenation
ICU: intensive care unit
LYM: lymphocyte
PCR: polymerase chain reaction
PEEP: positive end‐expiratory pressure

## 1 INTRODUCTION

Influenza is estimated to cause three to five million cases of severe illness and approximately 290 000–650 000 deaths each year, according to the World Health Organization statistics. The 2017 Global Burden of Disease Study estimated that lower respiratory tract infections caused by influenza resulted in 945 900 hospitalizations and 145 000 deaths in all age groups.^1^ In recent years, it has been gradually recognized that influenza combined with bacterial infection and Aspergillus infection are risk factors for poor prognosis of influenza.^2^, ^3^, ^4^ However, few studies have concentrated on coinfection of influenza with other viruses.

Epstein–Barr virus (EBV) belongs to human herpesvirus type,^4^ and EBV infection was reported in more than 90% of the global population.^5^ Chronic infection or isolation with EBV is associated with several malignant diseases, including lymphoma and nasopharyngeal carcinoma.^6^ Studies have shown that when EBV is combined with other viruses, the disease may be more serious.^7^, ^8^, ^9^ In the past 2 years, studies have also reported that EBV rekindled in patients with severe coronavirus disease 2019 (COVID‐19) infection and affected their prognosis.^10^, ^11^ In clinical practice, we also found the isolation of EBV in lower respiratory tract specimens of a certain proportion of patients with influenza A pneumonia. Whether the conditions of these patients are more serious, and whether the isolation of EBV in the lower respiratory tract indicates a poor prognosis of the patients, have not been previously discussed. Hence, this retrospective study aimed to explore clinical impact of EBV‐DNA in the lower respiratory tract, in order to differentiate critically ill patients from those with influenza‐associated pneumonia.

## 2 MATERIALS AND METHODS

### 2.1 Study design

This retrospective observational study was conducted at Beijing Chao‐Yang Hospital Affiliated to Capital Medical University (Beijing, China). The data of cases included in this study were derived from the baseline data of a clinical observation cohort of hospitalized patients with influenza A‐related pneumonia. This study was approved by the Institutional Review Board of Beijing Chao‐Yang Hospital Affiliated to Capital Medical University (Approval No. 2015‐KE‐158). Due to the use of data anonymization process, informed consent was waived with the approval of the aforementioned institutional review board.

### 2.2 Study population

This retrospective study enrolled patients with influenza A‐related pneumonia who were hospitalized in the Department of Respiratory and Critical Care Medicine and the Department of Infectious Diseases and Clinical Microbiology, Beijing Chao‐Yang Hospital Affiliated to Capital Medical University for six influenza seasons from the 2013–2014 influenza season to the 2018–2019 influenza season. The inclusion criteria were as follows: (1) age ≥18 years old; (2) positivity of influenza A virus was confirmed by influenza virus antigen test or polymerase chain reaction (PCR) test; and (3) consistency with the diagnosis of pneumonia. In addition, two radiologists independently reviewed the images and judged that they were consistent with influenza pneumonia. The exclusion criteria were as follows: (1) patients who did not provide lower respiratory tract specimens (qualified sputum or bronchoalveolar lavage fluid) for detection of EBV using PCR test within 3 days of being admitted and (2) incomplete clinical data.

### 2.3 Data collection

The clinical data of this study were obtained from the baseline data of the abovementioned retrospective cohort, including medical visits, demographic data, clinical manifestations, laboratory tests, and treatment. Among them, the visiting conditions and demographic data included age, gender, underlying diseases, time from onset to admission, length of hospital stay, and survival to discharge rate. Clinical data included history of undergoing mechanical ventilation, history of undergoing continuous renal replacement therapy, and intensive care unit (ICU) admission. Laboratory tests comprised blood routine test, blood gas analysis, liver and kidney function, albumin, coagulation function, inflammatory biomarkers, counting of T cell subsets, sputum bacteria and mycological results, blood culture, respiratory virus nucleic acid detection, and so forth.

All patients with influenza A‐related pneumonia included in the study had lower respiratory tract specimens and nucleic acid detection of respiratory viruses, including EBV, for examination upon admission (within 3 days). EBV‐DNA was detected through real‐time PCR (Kit: Liferiver, Z‐OD‐0023‐02).

Acute respiratory distress syndrome (ARDS) was defined as noncardiogenic pulmonary edema manifesting as acute onset, bilateral lung infiltrates on imaging, respiratory failure unexplained by heart failure, or fluid overload. ARDS was graded according to the arterial oxygen partial pressure (PaO_2_)/fraction of inspired oxygen (FiO_2_) ratio: mild ARDS: 200 mmHg < PaO_2_/FiO_2_ ≤ 300 mmHg and positive end‐expiratory pressure (PEEP) or continuous positive airway pressure ≥ 5 cmH_2_O; moderate ARDS: 100 mmHg < PaO_2_/FiO_2_ ≤ 200 mmHg and PEEP ≥ 5 cmH_2_O; and severe ARDS: PaO_2_/FiO_2_ ≤ 100 mmHg and PEEP ≥ 5 cmH_2_O. In this study, patients with mild ARDS and those who did not meet the criteria for ARDS were classified into the mild group, and patients with moderate and severe ARDS were classified into the severe group.

### 2.4 Statistical analysis

Statistical analysis was performed using SPSS 23.0 software (IBM Inc., Armonk, NY, USA). Data were presented as either median with interquartile range or mean ± standard deviation for numerical variables and count and percentage for categorical variables, as appropriate. Continuous variables with normal distribution were compared using the *t*‐test, while those with nonnormal distribution were compared using the Mann–Whitney U test. Categorical variables were compared using the Chi‐square test. Variables that differed significantly between severe and mild groups were considered as potential risk factors. Univariate and multivariate logistic regression analyses were performed to screen the risk factors for influenza A‐related pneumonia. The results were presented as estimates of relative risk by odds ratio (OR) with a 95% confidence interval (CI). Statistical significance was defined at *P* < 0.05.

## 3 RESULTS

### 3.1 Patients with influenza A‐related pneumonia who were found the isolation of EBV experienced a more serious illness

A total of 113 cases met the criteria, including 53 patients with EBV‐positive in lower respiratory tract specimens and 60 patients with EBV‐negative.

The relative number of EBV‐positive patients over different years was shown in Table 1. The proportion of EBV isolated in patients with influenza A‐related pneumonia showed an increasing trend, but there was no significant difference in the number of EBV isolated in different years.

**TABLE 1:** Relative number of EBV‐positive and EBV‐negative patients over different years.

Years	Whole population (*n* = 113)	EBV‐positive group (*n* = 53)	EBV‐negative group (*n* = 60)	*P‐*value
2013–2014	11 (9.73%)	5 (9.43%)	6 (10.00%)	0.190
2014–2015	2 (1.77%)	0	2 (3.33%)	
2015–2016	7 (6.19%)	2 (3.77%)	5 (8.33%)	
2016–2017	21 (18.58%)	10 (18.87%)	11 (18.33%)	
2017–2018	32 (28.32%)	20 (37.74%)	12 (20.00%)	
2018–2019	40 (35.40%)	16 (30.19%)	24 (40.00%)	

Abbreviation: EBV, Epstein–Barr virus.

The positive rate of EBV‐DNA in the lower respiratory tract of hospitalized patients with influenza A‐related pneumonia was 46.9%. The demographic characteristics, clinical visits, and prognostic data of the two groups of patients are presented in Table 2.

**TABLE 2:** Demographic characteristics, clinical visits, and prognostic data of patients in EBV‐positive and EBV‐negative groups.

	Whole population (*n* = 113)	EBV‐positive group (*n* = 53)	EBV‐negative group (*n* = 60)	*P‐*value
Age (years)	51.21 ± 15.56	52.55 ± 14.38	50.03 ± 16.56	0.394
Gender (male/female)	1:0.41	1:0.29	1:0.54	0.151
Underlying disease, no. (%)				
Hypertension	38 (33.63%)	15 (28.30%)	23 (38.33%)	0.260
Diabetes mellitus	19 (16.81%)	9 (16.98%)	10 (16.67%)	0.852
Chronic lung disease	14 (12.39%)	3 (5.66%)	11 (18.33%)	0.079
Immunocompromised^a^	11 (9.73%)	5 (9.43%)	6 (10.00%)	0.920
Coronary heart disease	8 (7.08%)	5 (9.43%)	3 (5.00%)	0.583
Renal insufficiency	8 (7.08%)	5 (9.43%)	3 (5.00%)	
Time from onset of symptoms to hospital admission (days)	7.82 ± 5.08	8.26 ± 6.02	7.43 ± 4.09	0.388
Length of hospital stay (days)	14 (7.5, 27)	16 (9, 32.5)	13 (6, 21.75)	0.039
Ventilatory strategies, no. (%)				
Noninvasive ventilation	35 (30.97%)	19 (35.84%)	16 (26.67%)	0.294
Invasive mechanical ventilation	68 (60.18%)	37 (69.81%)	31 (51.67%)	0.050
ECMO	30 (26.55%)	20 (37.74%	10 (16.67%)	0.012
CRRT, no. (%)	21 (21.00%)	13 (25.49%)	8 (16.33%)	0.263
ICU admission, no. (%)	81 (71.68%)	43 (81.13%)	38 (63.33%)	0.037
SOFA	6.70 ± 4.31	7.66 ± 3.79	5.85 ± 4.58	0.025
APACHE‐II	11.32 ± 4.52	11.60 ± 4.62	11.07 ± 4.46	0.531
Moderate‐to‐severe ARDS,^b^ no. (%)	73 (64.60%)	41 (77.36%)	32 (53.33%)	0.008
Death, no. (%)	35 (30.97%)	17 (32.08%)	18 (30.00%)	0.813

Abbreviations: APACHE‐II, Acute Physiology and Chronic Health Evaluation II; ARDS, acute respiratory distress syndrome; CRRT, continuous renal replacement therapy; EBV, Epstein‐Barr virus; ECMO, extracorporeal membrane oxygenation; ICU, intensive care unit; SOFA, Sequential Organ Failure Assessment.

^a^ Including post‐organ transplant, patients with hematological, solid tumor, and immune system diseases.

^b^ Moderate‐to‐severe ARDS according to the Berlin Standard: positive end‐expiratory pressure ≥ 5 cmH_2_O and arterial oxygen partial pressure/fraction of inspired oxygen ratio ≤ 200 cmH_2_O.

In the EBV‐positive group, the median hospital stay was longer, the proportion of ICU admission was higher, more patients received invasive mechanical ventilation and extracorporeal membrane oxygenation (ECMO), and Sequential Organ Failure Assessment (SOFA) score and the rate of progression to moderate‐to‐severe ARDS was higher, in which the differences between the two groups were statistically significant. The proportion of patients in the EBV‐positive group requiring continuous renal replacement therapy for renal replacement therapy was higher than that in the EBV‐negative group, while the difference was not statistically significant. There were no significant differences in age, sex, underlying diseases, APACH‐II scores, and mortality between the two groups.

### 3.2 The isolation of EBV could be one of the laboratory markers for patients with severe influenza A‐related pneumonia

In order to further analyze the meaning of EBV isolation on patients with influenza A‐related pneumonia, patients with influenza A‐related pneumonia were regrouped according to the severity of the disease. Patients who met the criteria for moderate‐to‐severe ARDS were involved in the severe group, and other patients were included in the mild group.

The differences in laboratory parameters between the severe group and the mild group are shown in Table 3. Compared with the mild group, the lymphocyte (LYM) count, platelet count, and albumin level in the severe group were lower, and the difference between the two groups was statistically significant. The levels of aspartate aminotransferase, alanine transaminase, creatine kinase, lactic dehydrogenase, total bilirubin, D‐dimer, serum ferritin, C‐reactive protein, and procalcitonin in the severe group were higher than those in the mild group, and the difference was statistically significant. In addition, the positive rate of EBV in lower respiratory tract specimens, the incidence of bacterial infection, and bacteremia in the severe group were higher than those in the mild group, and the differences were statistically significant.

**TABLE 3:** Comparison of laboratory results among different groups.

	Severe group (*n* = 73)	Mild group (*n* = 40)	*P‐*value
White blood cells (×10^9^/L)	6.90 ± 3.83	6.25 ± 5.95	0.481
Lymphocytes (×10^9^/L)	0.63 ± 0.36	0.96 ± 0.60	1
Platelet (×10^9^/L)	138.70 ± 65.10	178.90 ± 92.32	0.008
Albumin (g/L)	30.60 ± 5.02	34.14 ± 5.91	0.001
Aspartate aminotransferase (U/L)	67.00 (44.00, 133.00)	37.00 (25.50, 52.25)	<0.001
Alanine transaminase (U/L)	33.00 (22.50, 65.50)	24.00 (17.25, 42.00)	0.008
Creatine kinase (U/L)	198.00 (99.00, 503.00)	132.50 (53.25, 276.25)	0.012
Lactic dehydrogenase (U/L)	773.81 ± 550.66	417.00 ± 252.64	<0.001
Total bilirubin (μmol/L)	12.60 (8.20, 19.20)	8.65 (5.68, 17.98)	0.048
Blood urea nitrogen (mmol/L)	8.49 ± 5.39	7.50 ± 6.15	0.374
Creatinine (μmol/L)	103.42 ± 89.17	93.08 ± 78.66	0.541
D‐Dimer (mg/dL)	4.59 (2.31, 14.07)	1.73 (0.96, 2.96)	<0.001
Serum ferritin (mg/dL)	1745.20 (986.40, 3042.20)	786.00 (274.95, 1377.28)	<0.001
CRP (mg/L)	12.00 (7.92, 19.10)	4.54 (1.11, 9.42)	<0.001
IgG (mg/L)	906.14 ± 359.02	791.52 ± 405.91	0.164
Complement 3 (mg/dL)	67.69 ± 25.17	73.29 ± 33.65	0.361
Complement 4 (mg/dL)	23.16 ± 11.22	24.15 ± 10.54	0.684
CD4^+^ T cells (/μL)	176.00 (115.50, 298.00)	241.00 (87.00, 414.00)	0.385
CD8^+^ T cells (/μL)	93.50 (68.75, 152.50)	123.00 (70.00, 241.00)	0.148
Procalcitonin (ng/dL)	1.03 (0.34, 6.43)	0.18 (0.05, 0.68)	<0.001
EBV‐positive, no. (%)	41 (56.16%)	12 (30.00%)	0.008
Coinfection with bacteria, no. (%)	43 (58.90%)	5 (12.50%)	<0.001
Coinfection with fungi, no. (%)	36 (49.32%)	15 (37.50%)	0.111
Coinfection with other virus,^a^ no. (%)	8 (10.96%)	4 (10.00%)	1.000
Bacteremia, no. (%)	12 (16.43%)	1 (2.50%)	0.013

Abbreviations: ADV, adenovirus; CMV, cytomegalovirus; CRP, C‐reactive protein; EBV, Epstein–Barr virus; HRV, human rhinovirus; IgG, Immunoglobulin G; RSV, respiratory syncytial virus.

^a^ Including eight cases of CMV positive, two cases of RSV positive, one case of ADV positive, and one case of HRV positive.

### 3.3 The isolation of EBV‐DNA could be an independent risk factor for moderate‐to‐severe ARDS in patients with influenza A‐related pneumonia

Variables that were meaningful in the comparison between the severe and mild groups were included in multivariate logistic regression analysis to find independent risk factors for moderate‐to‐severe ARDS in patients with influenza A‐related pneumonia. The results showed that the isolation of EBV‐DNA (OR = 2.713, 95% CI: 1.094–6.729, *P* = 0.031) and LYM count (OR = 3.585, 95% CI: 1.157–11.101, *P* = 0.027) were associated with moderate‐to‐severe ARDS in patients with influenza A‐related pneumonia.

## 4 DISCUSSION

In this observational study, based on baseline data from a single‐center retrospective cohort, the relationship between EBV isolation and hospitalized patients with influenza A‐related pneumonia was clarified. Positive EBV‐DNA results in lower respiratory tract specimens were found as independent risk factors for the development of moderate‐to‐severe ARDS in hospitalized patients with influenza A‐related pneumonia. This is the first evaluation of the relationship between isolation of EBV and disease severity in patients with influenza A‐related pneumonia.

The results of the present study showed that the isolation rate of EBV‐DNA in the lower respiratory tract specimens of patients with influenza A‐related pneumonia was 46.9%. Although the flu virus strains differ from year to year, which may have an impact on the results, there was no significant difference in the number of EBV isolated in different years. It has been reported that the application of PCR method to detect EBV in the lower respiratory tract specimens of patients with several respiratory diseases was accompanied by a high detection rate, in which detection rates of chronic obstructive pulmonary disease and bronchiectasis were 46–48% and 48.1%, respectively, and continued detection of EBV was found to be associated with the disease progression.^12^, ^13^ The lower respiratory tract is the main site of EBV in the incubation period.^14^ The life cycle of EBV includes the dissolution period and the incubation period, which are involved in the spread of the virus and the maintenance of the genome.^15^ More than 90% of the global adult population has been reported to be infected with EBV^5^. Therefore, the isolation of EBV‐DNA in the lower respiratory tract in adults is more likely to be due to the colonization of the respiratory tract or a previous infection that some reasons caused their DNA to be released from such as LYM, rather than a new infection. The specific mechanism in which EBV fluctuates between the dissolution period and the incubation period to maintain balance with the host is still unclear. EBV is highly such an opportunistic pathogen and reignites under some appropriate conditions. It has been reported that EBV reactivation is assisted by CD8^+^ T cells, CD4^+^ T cells, and natural killer cells.^16^ In addition, it has also been found that EBV is easily activated when the immune function declines in some clinical studies.^17^, ^18^ However, in the present study, it was revealed that there was no significant difference between the two groups in underlying diseases combined with immunocompromised and diabetes mellitus. The detection rate of EBV‐DNA was not correlated with patients' immune status, which could be related to the general susceptibility of influenza A virus population and the fact that nonimmunodeficient patients had the highest proportion people, and only 9.73% of patients had immunodeficiency disorders. In addition, there may be some promoting mechanism for the increase of EBV detection rate caused by acute influenza virus infection, which has nothing to do with the underlying immune status of the body.

A study of EBV‐DNA detection in bronchoalveolar lavage of solid organ transplant patients indicated that EBV‐DNA was frequently detected in patients with pneumonia, respiratory insufficiency, and other acute exacerbations of bronchopulmonary disease, although the isolation rate of EBV‐DNA in the transplant group was higher than that in the nontransplant group. However, the presence of EBV was not associated with the increased 28 day mortality.^19^ It is noteworthy that EBV can be such an indicator of the degree of airway inflammatory damage, and EBV is easier to be found in patients with severe damage. The present study revealed that patients with influenza A‐related pneumonia with isolation of EBV‐DNA were more severely ill in terms of respiratory inflammatory damage, which mainly reflected in moderate‐to‐severe ARDS and higher rates of invasive mechanical ventilation and ECMO use. There was no significant difference in mortality between the two groups.

Codetection of EBV and some viruses can exacerbate diseases.^7^, ^8^, ^9^ In some mechanistic studies, herpesvirus codetection was shown to affect immune cell dynamics, such as formation and activity of natural killer cells^20^, ^21^ and expansion and activity of B cells.^22^ However, the relationship between influenza virus and EBV has not yet been reported. In order to further analyze whether the isolation of EBV is associated with the exacerbation of influenza A‐related pneumonia, patients with influenza A‐related pneumonia were divided into two groups according to the disease conditions. The results were partly similar to those reported previously.^2^, ^23^, ^24^, ^25^ In this study, there were 12 patients who tested positive for influenza A and EBV and had positive results for other respiratory virus, but there was no statistical difference in coinfection between mild and severe groups. The LYM count, platelet count, and albumin level in the severe group were lower; the levels of aspartate aminotransferase, alanine transaminase, creatine kinase, lactic dehydrogenase, D‐dimer, C‐reactive protein, procalcitonin, and so forth in the severe group were higher than those in the mild group. Bacterial infection and bacteremia rate were both higher in the severe group.

This study further indicated that the low LYM count and the isolation of EBV were independent risk factors for the development of moderate‐to‐severe ARDS in patients with influenza A‐related pneumonia. A previous study demonstrated that decreased LYM count was an independent risk factor for death from influenza‐related pneumonia,^25^ which was similar to our findings. There are some recent reports on blood EBV reactivation in patients with COVID‐19, and they mainly reported that there is a certain level of EBV reactivation in blood of patients with severe COVID‐19 infection, while it could not be confirmed that EBV hyperemia is caused by COVID‐19 infection. It was found to be remarkably correlated with disease severity.^10^, ^11^, ^26^ The relationship between EBV and mortality is inconsistent,^10^, ^11^ and in a small study, EBV‐DNA was detected in the blood of 28 of 34 patients hospitalized with respiratory failure due to COVID‐19 infection, and EBV reactivation was associated with the longer ICU stay, rather than with mortality.^10^ Chinese scholars demonstrated that COVID‐19 patients with EBV reactivation had a higher mortality rate than non‐EBV‐infected patients.^11^ The abovementioned studies on COVID‐19 all used the EBV‐DNA‐positive in blood to define the EBV reactivation rate to evaluate the disease, which may underestimate the reactivation rate of EBV in the respiratory tract, and there is a lack of reports on the correlation between EBV and prognosis in patients with influenza A‐related pneumonia. This is the first research to demonstrate that the isolation of lower respiratory tract EBV‐DNA was associated with the severity of influenza A‐related pneumonia, and it was noted as an independent risk factor for moderate‐to‐severe ARDS, while it was not associated with mortality.

This study has some limitations. First, this is a retrospective study. Therefore, the data of EBV antibody and DNA copy number in the blood samples of the majority of patients could not be obtained, and quantitative PCR analysis of respiratory specimens was not performed; thus, it could not be better explained that these patients were EBV reactivated or colonized. This study only shows that isolation of EBV in the lower respiratory tract can be used as a marker of severe illness in patients with influenza pneumonia. Second, noninfluenza A‐related pneumonia patients were not included as a comparison population, disabling us to indicate whether influenza virus infection was also a driver of EBV positivity. However, further attention should be paid to the conclusions of this study by clinicians in the next studies.

In conclusion, the isolation of EBV‐DNA in the lower respiratory tract of in hospitalized patients with influenza A‐related pneumonia was 46.9%. Besides, moderate‐to‐severe ARDS is more frequent in patients with lower respiratory tract of EBV‐DNA‐positive influenza A‐related pneumonia, and the SOFA score and the proportions of patients undergoing invasive mechanical ventilation and ECMO were higher. The isolation of EBV and low LYM count were found as independent risk factors for moderate‐to‐severe ARDS in hospitalized patients with influenza A‐related pneumonia.

## AUTHOR CONTRIBUTIONS

Li Gu and Yu Bai play a guiding role in designing the study and responsible for adding new data. Yu Bai also mainly takes charge of statistical analysis of all data and revision of manuscript. Yiqun Guo performed original data collection and wrote the original manuscript. Xi Chen and Chunxia Yang was responsible for data analyzing and recruiting the patients. All authors read and approved the final manuscript.

## CONFLICT OF INTEREST STATEMENT

The authors declare no competing interests.

## ETHICS STATEMENT

The study was approved by the Institutional Review Board of Beijing Chao‐Yang Hospital. As the anonymized data in the current retrospective study was used, a waiver of informed consent was included in the approval from the Institutional Review Board of Beijing Chao‐Yang Hospital. All methods in this study were carried out in accordance with relevant guidelines and regulations (declarations of Helsinki).

## Data Availability

The deidentified data and statistical analysis code that support the findings of this study are available on reasonable request from the corresponding author.
